# Lectures on Tropical Diseases

**Published:** 1906-03

**Authors:** 


LECTURES ON TROPICAL DISEASES.—Being the Lane lectures for 1905, delivered at Cooper Medical College, San Francisco, Cal., August, 1905. By Sir Patrick Manson, K. C. M. G., M. D., LL. D. (Aberdeen), F. R. C. P. (London), F. R. S., Hon. D. Sc. (Oxon), Medical Adviser to the Colonial Office, Lecturer London School of Tropical Medicine, St. George's Hospital Medical School, etc., etc. Chicago: W. T. Keener & Co., 90 Wabash Ave., 1905. Cloth bound, 230 pages; price, $2.50, net.

This book, in its mechanical get-up, is a fine specimen of art. It is as neat a piece of bookmaking as we have seen. The lectures cover a wide range. Part I deals with "Principles Determining Geographical Distribution of Tropical Diseases: Ephiphitic Disease: Ankylostomiasis." It is illustrated with the pictures of many of the tinea affecting man, and is in this regard especially interesting, in as much as great attention is now being paid to such factors in disease. Part II, "Dracontiasis: Endemic Haemoptysis." Part III, "Bilharziosis: Filariasis"; illustrated and very interesting, as indeed are all the subjects treated.

This is something new. Send for it. The chapter on "The Sleeping Sickness," is especially interesting. D.

